# In Vivo Transcriptomics-Based Framework for Systematic Indication Expansion

**DOI:** 10.64898/2026.10.12.758524

**Published:** 2026-10-14

**Authors:** Zsolt Palmer, Shubham Koirala, Nabasmita Talukdar, Xiaodan Zhang, Hannah Garver, McKenzie Mahlmeister, Li Huang, Dimitri Joseph, Dmitry Leshchiner, Rama Shankar, Stephanie W. Watts, Xiaopeng Li, Benjamin S. Glicksberg, Adam Lauver, Bin Chen

## Abstract

Capturing the systemic effects of drugs *in vivo* remains a fundamental challenge for drug repurposing strategies. Here, we present an *in vivo* transcriptomics-based drug repurposing framework that profiles multi-organ RNA-seq from drug-treated rodents to model systemic drug responses and match tissue-specific drug signatures against a compendium of disease signatures for indication expansion. We applied this framework to semaglutide, a GLP-1 receptor agonist originally developed for diabetes and subsequently expanded for several other indications. By harmonizing more than 900 human disease transcriptomic signatures via agentic AI and using Z-transformed reversal of gene expression scoring to quantify drug-disease relationships, our approach successfully recovered established semaglutide indications while deprioritizing Alzheimer’s disease, an indication for which semaglutide was not effective. Top predictions for common diseases were supported by real-world electronic health record data from the Mount Sinai Health System and several predictions for rare indications are proposed for further investigation. This framework provides a scalable strategy for indication expansion across diverse therapeutic agents, especially investigational compounds for which real-world evidence remains sparse.

**One Sentence Summary:**

Comparing drug and disease expression profiles from *in vivo* contexts achieves indication profiles reflective of the interconnected mammalian organism.

